# Efficient 2,3-butanediol production from whey powder using metabolically engineered *Klebsiella oxytoca*

**DOI:** 10.1186/s12934-020-01420-2

**Published:** 2020-08-10

**Authors:** Wensi Meng, Yongjia Zhang, Menghao Cao, Wen Zhang, Chuanjuan Lü, Chunyu Yang, Chao Gao, Ping Xu, Cuiqing Ma

**Affiliations:** 1 State Key Laboratory of Microbial Technology, Shandong University, Qingdao, 266237 People’s Republic of China; 2 Center for Gene and Immunotherapy, The Second Hospital of Shandong University, Jinan, 250033 People’s Republic of China; 3 State Key Laboratory of Microbial Metabolism, Joint International Research Laboratory of Metabolic & Developmental Sciences, and School of Life Sciences & Biotechnology, Shanghai Jiao Tong University, Shanghai, 200240 People’s Republic of China

**Keywords:** Whey, Lactose, *Klebsiella oxytoca* PDL-0, 2,3-Butanediol, Metabolic engineering

## Abstract

**Background:**

Whey is a major pollutant generated by the dairy industry. To decrease environmental pollution caused by the industrial release of whey, new prospects for its utilization need to be urgently explored. Here, we investigated the possibility of using whey powder to produce 2,3-butanediol (BDO), an important platform chemical.

**Results:**

*Klebsiella oxytoca* strain PDL-0 was selected because of its ability to efficiently produce BDO from lactose, the major fermentable sugar in whey. After deleting genes *pox*, *pta*, *frdA*, *ldhD*, and *pflB* responding for the production of by-products acetate, succinate, lactate, and formate, a recombinant strain *K. oxytoca* PDL-K5 was constructed. Fed-batch fermentation using *K. oxytoca* PDL-K5 produced 74.9 g/L BDO with a productivity of 2.27 g/L/h and a yield of 0.43 g/g from lactose. In addition, when whey powder was used as the substrate, 65.5 g/L BDO was produced within 24 h with a productivity of 2.73 g/L/h and a yield of 0.44 g/g.

**Conclusion:**

This study demonstrated the efficiency of *K. oxytoca* PDL-0 for BDO production from whey. Due to its non-pathogenicity and efficient lactose utilization, *K. oxytoca* PDL-0 might also be used in the production of other important chemicals using whey as the substrate.

## Background

Whey, a liquid by-product generated during cheese production, contains most of the water-soluble components in milk [1, 2]. Despite annual production of 145 million tons worldwide, only a little over one-half of the whey produced is utilized [3]. Whey is regarded as a serious pollutant because of its high biochemical oxygen demand (BOD, 30,000–50,000 mg/L) and chemical oxygen demand (COD, 60,000–80,000 mg/L) [3]. Economic disposal of whey has become a worldwide problem for the dairy industry. Lactose, a utilizable disaccharide for many microbial strains, is the major contributor to BOD and COD of whey [4, 5]. Using the lactose in whey as a substrate for industrial microbial fermentation may transform a potential pollutant into a value-added product and this prospect deserves an intensive study.

2,3-Butanediol (BDO) is an important platform chemical that can be applied in many industrial fields [6–8]. Derivatives of BDO are estimated to have a potential global market of around 32 million tons per year. One common method for BDO synthesis is performed under harsh conditions (160–220 °C, 50 bar) with a C_4_ hydrocarbon fraction of cracked gases as the substrate [9, 10]. However, due to shortage of fossil fuels and increasing global environmental concerns, green production of BDO through microbial fermentation is desirable [11–16]. Renewable resources such as rice waste biomass, sugarcane bagasse hydrolysate, and kenaf core biomass have been used in fermentative production of BDO [17–19].

Several BDO-producing microorganisms can use fermentable sugars, including glucose, xylose, fructose, and lactose as the sole carbon source for growth [20–23]. However, these strains exhibit unsatisfactory fermentative performance in BDO production when lactose is used as the carbon source. For example, *Klebsiella oxytoca* NRRL-B199 can use the mixture of glucose and galactose as substrate for growth and produce BDO as its main product. Nevertheless, BDO was present in a low concentration and the strain produced acetate as the major product in the fermentation broth with lactose [24, 25].

**Fig. 1:**
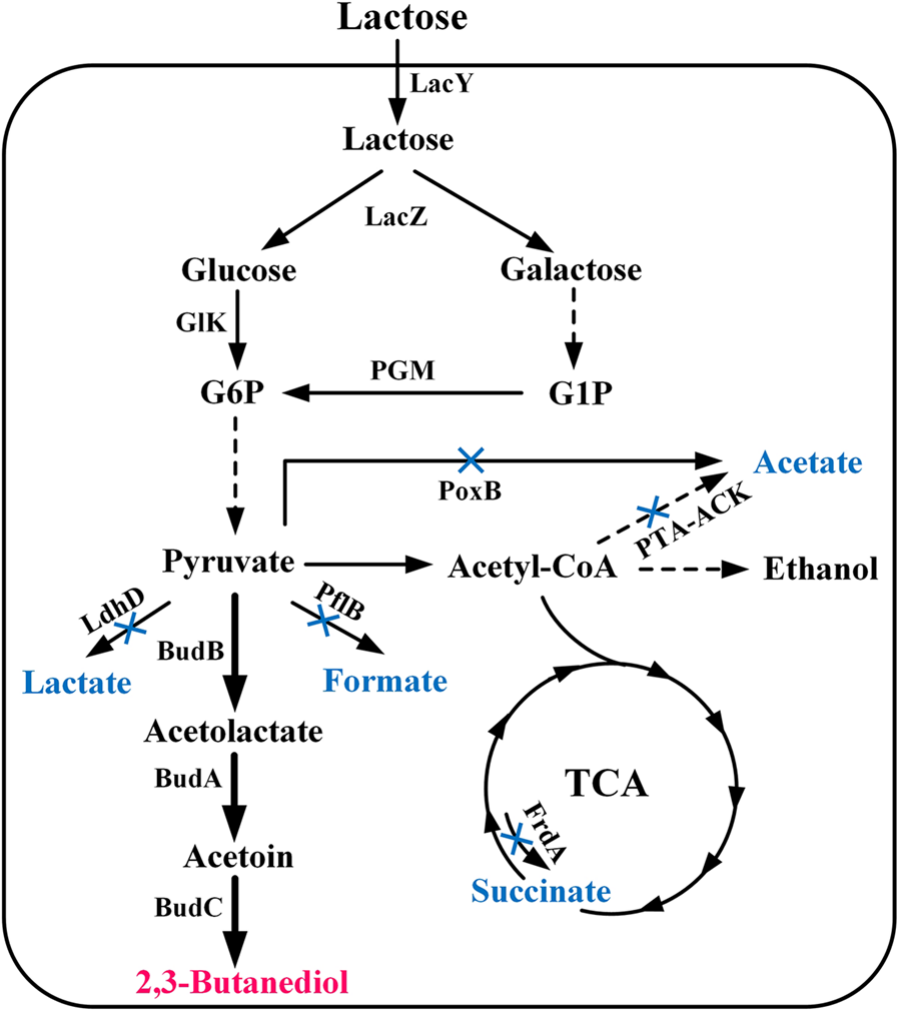
Metabolic engineering strategies for efficient production of BDO from whey powder by *K. oxytoca* PDL-0. Solid lines represent one step reactions. Dashed lines represent multi-step reactions. Blue crosses indicated the blocked pathways in the metabolic engineered strain. The target product is shaded in red and the blocked byproducts are shaded in blue. *G1P* glucose-1-phosphate, *G6P* glucose-6-phosphate, *LacY* lactose permease, *LacZ* β-galactosidase, *GlK* glucose kinase, *PGM* phosphoglucomutase, *PoxB* pyruvate oxidase, *PTA* phosphotransacetylase, *ACK* acetate kinase, *FrdA* catalytic subunit of fumarate reductase, *LdhD* lactate dehydrogenase, *PflB* pyruvate formate-lyase, *BudB* α-acetolactate synthase, *BudA* α-acetolactate decarboxylase, *BudC* acetoin reductase

Production of BDO using whey as the substrate can enhance the economic feasibility of BDO fermentation and facilitate resource utilization of the pollutant whey. Therefore, it is critical to identify a suitable microbial strain with BDO production potential using lactose and whey. In this study, we cultured *Klebsiella pneumonia* ATCC 15380, *Enterobacter cloacae* SDM, *Bacillus licheniformis* DSM13, *K. oxytoca* PDL-0, and *Escherichia coli* BL21-pETRABC in fermentation broths with lactose as the carbon source. *K. oxytoca* PDL-0 exhibited the best performance in lactose utilization and BDO production. Next, byproduct-producing genes in *K. oxytoca* PDL-0, including *pox*, *pta*, *frdA*, *ldhD*, and *pflB*, were knocked out to improve the efficiency of BDO production from lactose. Finally, high production of BDO from whey powder was achieved through fed-batch fermentation using the recombinant strain (Fig. 1).

## Results and discussion

### Selection of *K. oxytoca* PDL-0 for BDO production from lactose

**Fig. 2:**
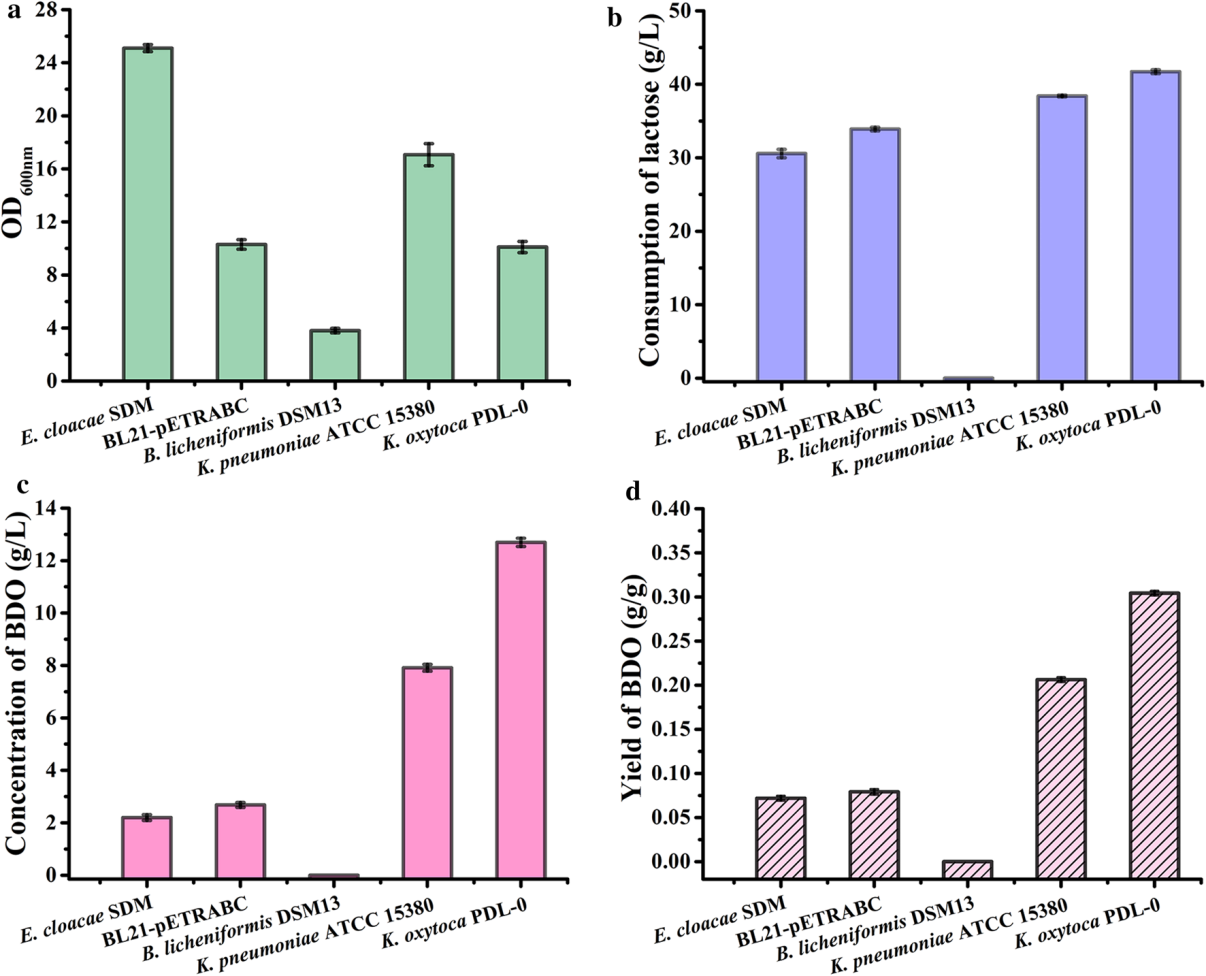
Selection for stains that can produce BDO from lactose. Biomass (**a**), consumption of lactose (**b**), concentration (**c**) and yield (**d**) of BDO using lactose as the carbon source by *E. cloacae* SDM, *E. coli* BL21-pETRABC, *B. licheniformis* DSM13, *K. pneumonia* ATCC 15380, and *K. oxytoca* PDL-0 were assayed. The experiments were conducted in a 300-mL flask containing 50 mL of M9 minimal medium supplemented with 5 g/L yeast extract and 40 g/L lactose with shaking at 180 rpm for 48 h. The culture temperature for *B. licheniformis* DSM13 was 50 °C while for other strains were 37 °C. The data for *K. oxytoca* PDL-0 and *K. pneumoniae* ATCC 15380 were obtained at 18 h and 36 h, respectively. The data for *E. cloacae* SDM, *B. licheniformis* DSM13 and *E. coli* BL21-pETRABC were obtained at 48 h. Error bars indicate the standard deviations from three independent cultures

To select a strain for efficient BDO production from whey, we first assessed strains that can utilize lactose and produce BDO. *K. pneumonia*, *E. cloacae*, *B. licheniformis*, and *K. oxytoca* can produce BDO from glucose [16]. *E. coli* BL21-pETRABC carrying the BDO pathway gene cluster from *E. cloacae* can also efficiently bio-transform glucose into BDO [26]. In the present study, we first compared the ability of *K. pneumonia* ATCC 15380, *E. cloacae* SDM, *B. licheniformis* DSM13, *K. oxytoca* PDL-0, and *E. coli* BL21-pETRABC to produce BDO from lactose; results are shown in Fig. 2.

All five strains were cultured in M9 medium supplemented with 5 g/L yeast extract and ~ 40 g/L lactose for 48 h. *B. licheniformis* DSM13 is the only strain that cannot consume lactose. *E. cloacae* SDM and *E. coli* BL21-pETRABC could grow well and utilize ~ 30 g/L lactose within 48 h, but only accumulated about 2 g/L BDO (Additional file 1: Fig. S1, Fig. 2a–c). *K. pneumonia* ATCC 15380 and *K. oxytoca* PDL-0 can completely consume ~ 40 g/L lactose within 36 h and 18 h, and produce BDO from lactose with a yield of 0.21 g/g and 0.30 g/g lactose, respectively (Additional file 1: Fig. S1 and Fig. 2d). Considering the fact that *K. oxytoca* PDL-0 belongs to Risk Group 1 [15] and produces BDO from lactose with a higher yield than other strains, this strain was selected for further study in successive experiments.

### Inactivation of by-product pathways in *K. oxytoca* PDL-0

**Fig. 3:**
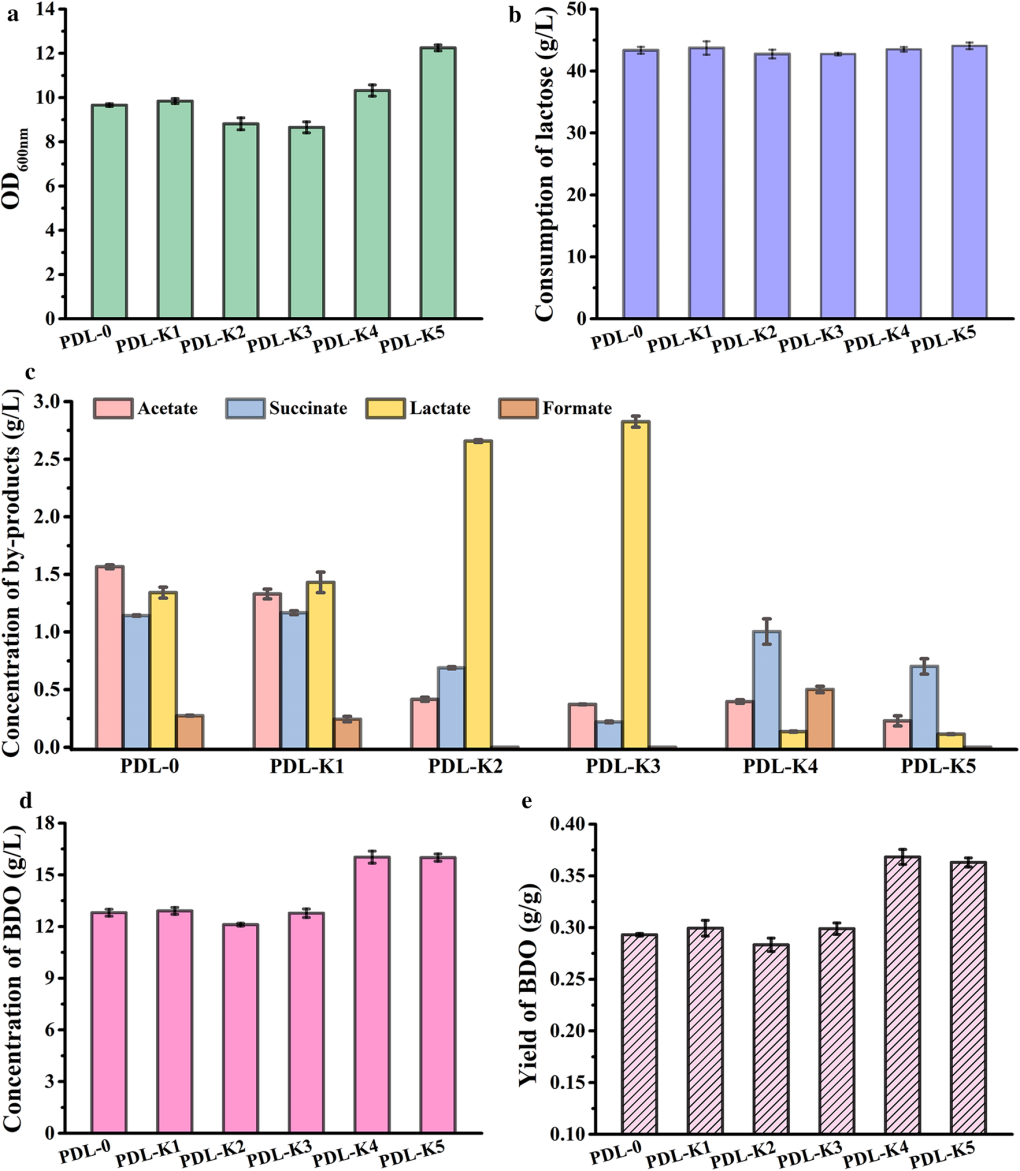
Effects of by-product pathway genes knockout when using lactose as the carbon source. Biomass (**a**), consumption of lactose (**b**), by-products (**c**), concentration (**d**) and yield (**e**) of BDO by *K. oxytoca* PDL-0 and its derivatives were assayed. The experiments were conducted in a 300-mL flask containing 50 mL of M9 minimal medium supplemented with 5 g/L yeast extract and 40 g/L lactose with shaking at 180 rpm for 24 h. The culture temperature was 37 °C. Error bars indicate the standard deviations from three independent cultures

*Klebsiella oxytoca* PDL-0 produced BDO as its major fermentative product during lactose fermentation in a shaking flask culture. However, only 56% of theoretical yield (0.293 vs 0.526 g/g) was observed (Fig. 3). BDO is produced by a fermentative pathway known as the mixed acid-BDO pathway in *K. oxytoca* [7, 15]. Acetate (1.57 g/L), succinate (1.14 g/L), lactate (1.34 g/L), and formate (0.27 g/L) were also detected as by-products in the fermentation broth (Fig. 3).

In *K. oxytoca* PDL-0, the formation of acetate, succinate, lactate, and formate is catalyzed by *pox* and *pta*, *frdA*, *ldhD*, and *pflB*, respectively [27]. To achieve higher BDO yield, these genes were successively deleted in strain *K. oxytoca* PDL-0 (Fig. 1). Effects of these gene deletions on growth, lactose consumption, by-product accumulation, and BDO production were studied in M9 medium supplemented with 5 g/L yeast extract and ~ 40 g/L lactose. As shown in Fig. 3a, b, deletion of these by-product pathways in *K. oxytoca* PDL-0 had no effect on lactose consumption but did slightly increase growth. Accumulation of by-products, including acetate (0.23 g/L), succinate (0.70 g/L), lactate (0.11 g/L), and formate (0 g/L), was markedly decreased due to deletion of *pox*, *pta*, *frdA*, *ldhD*, and *pflB* (Fig. 3c). The final strain, *K. oxytoca* PDL-K5, exhibited high concentration (16.0 g/L) and yield (0.36 g/g lactose) of BDO (Fig. 3d, e) and low by-product generation (Fig. 3c).

### Performance of recombinant strain in 1-L batch fermentation

**Fig. 4:**
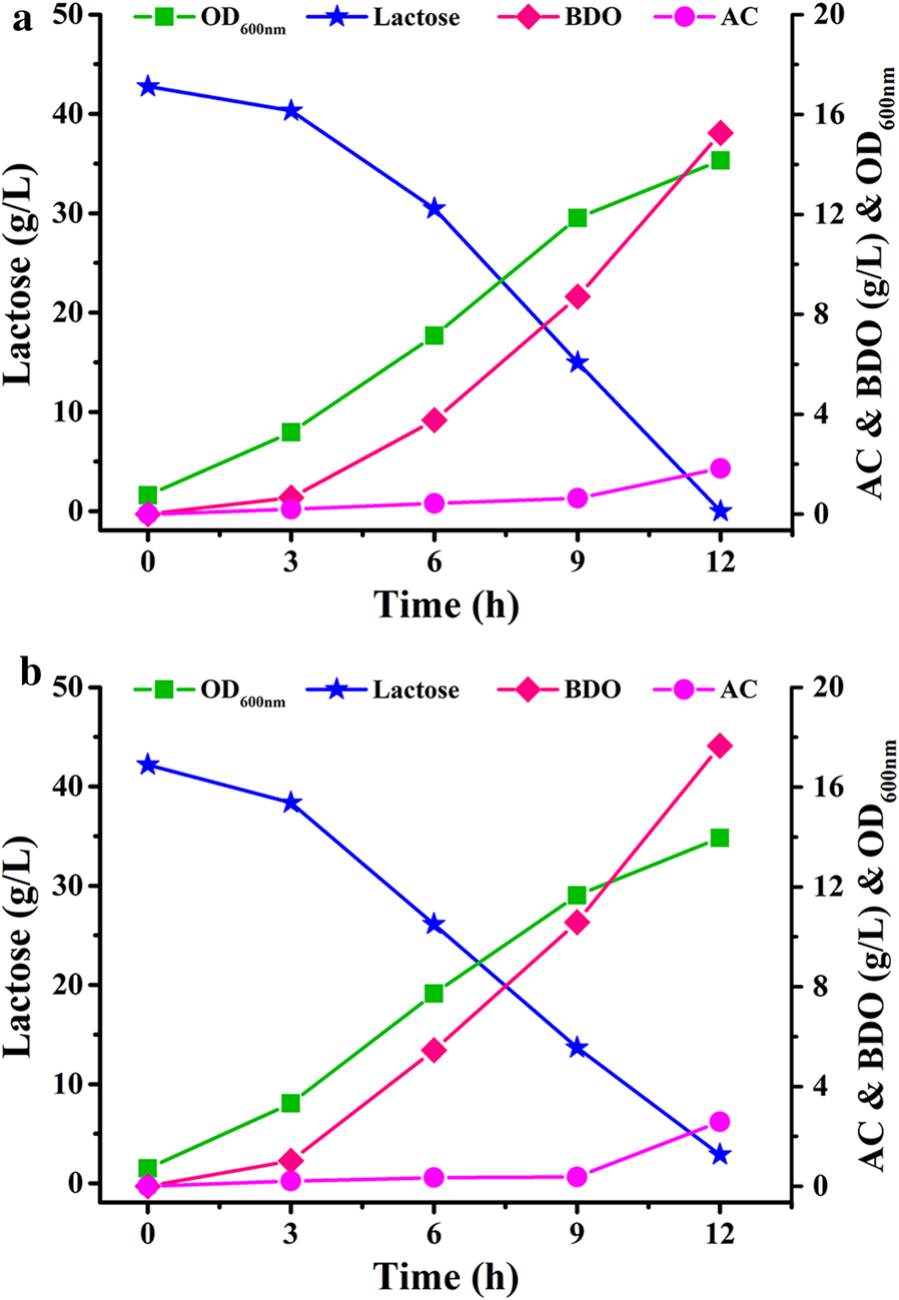
Batch fermentation using lactose as carbon source. Biomass, consumption of lactose, concentration of BDO and acetoin (AC) by *K. oxytoca* PDL-0 (**a**) and *K. oxytoca* PDL-K5 (**b**) were assayed. The experiments were conducted in a 1-L fermenter containing 800 mL of medium with an initial lactose concentration of 40 g/L approximately

The effects of inactivation of by-product pathways on BDO production were further studied through batch fermentation in a 1-L fermenter. The strains *K. oxytoca* PDL-0 and *K. oxytoca* PDL-K5 were cultured in a fermentation medium containing corn steep liquor powder as a nitrogen source and ~ 40 g/L lactose as carbon source. As shown in Fig. 4a, b, *K. oxytoca* PDL-0 consumed 42.75 g/L lactose and produced 15.26 g/L BDO with a yield of 0.36 g/g at 12 h, while *K. oxytoca* PDL-K5 consumed 39.29 g/L lactose and produced 17.65 g/L BDO with a yield of 0.45 g/g. Thus, the recombinant strain *K. oxytoca* PDL-K5 demonstrates advantages over wild type in both concentration and yield of BDO.

### Utilization of lactose for BDO production in fed-batch fermentation

**Fig. 5:**
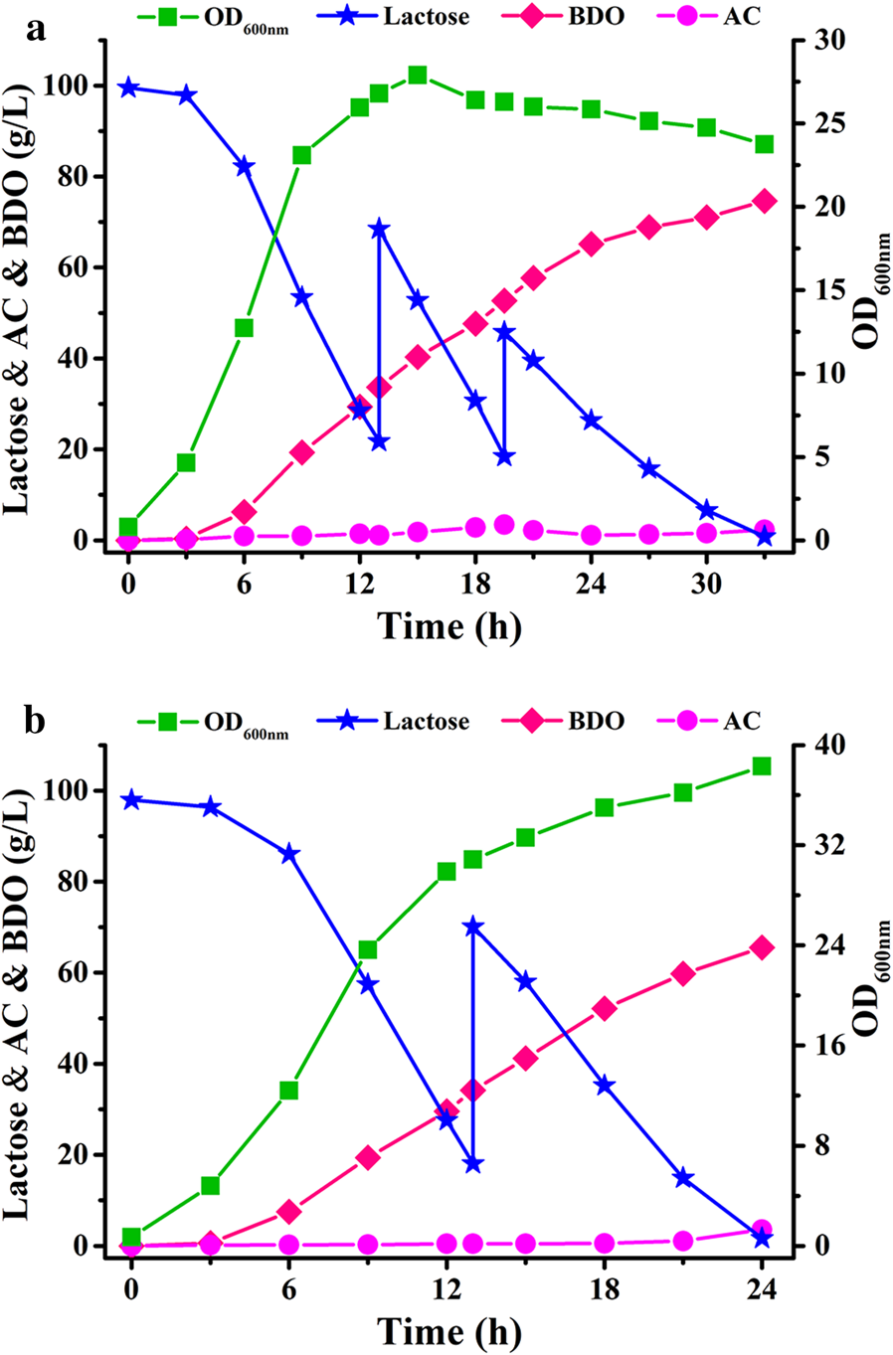
Fed-batch fermentation using lactose (**a**) and whey powder (**b**) as the carbon source. Biomass, consumption of lactose, concentration of BDO and acetoin (AC) by *K. oxytoca* PDL-K5 were assayed. The experiments were conducted in a 7.5-L fermenter containing 5 L of medium with an initial lactose concentration of 100 g/L approximately

To achieve higher product concentration, we performed fed-batch fermentation using strain *K. oxytoca* PDL-K5 with initial lactose concentration of ~ 100 g/L. Fermentation medium containing corn steep liquor was used in a 7.5-L fermenter. As shown in Fig. 5a, 173.2 g/L lactose was consumed and 74.9 g/L BDO was produced within 33 h. The productivity was 2.27 g/L/h and the yield was 0.43 g/g lactose. The final concentration of the major by-product succinate was 0.82 g/L and there was no formate production throughout the fermentation process (Additional file 1: Fig. S2a).

### Utilization of whey powder for BDO production in fed-batch fermentation

Fed-batch fermentation using *K. oxytoca* PDL-K5 with whey powder as the carbon source was also conducted. After 24 h of fermentation, 65.5 g/L BDO was obtained from 148.3 g/L lactose (Fig. 5b). The productivity and yield of BDO were 2.73 g/L/h and 0.44 g/g, respectively. The major by-products in the final fermentation broth were acetate and lactate, which were found at concentrations of 3.24 g/L and 0.38 g/L, respectively (Additional file 1: Fig. S2b). During fermentation, agitation and airflow were set at 400 rpm and 1 vvm, respectively, and dissolved oxygen was uncontrolled. Acetoin started to accumulate at the end of fermentation and feeding more whey powder into the fermentation system did not increase BDO production. Dissolved oxygen has a profound impact on the distribution of BDO and its dehydrogenation product, acetoin. Since BDO biosynthesis occurs under microaerobic conditions [28, 29], fine-tuning the dissolved oxygen through an automatic control system might provide the optimal microaerobic condition to further increase BDO production.

Several microbial strains have been screened to produce BDO from whey or lactose. However, as shown in Table 1, the final concentration and yield of BDO produced by wildtype isolates were relatively low. For example, Vishwakarma tried to use strain *K. oxytoca* NRRL-13-199 for BDO production from whey. After the addition of 50 mM acetate, 8.4 g/L BDO was acquired with a yield of 0.365 g/g lactose [30]. Barrett et al. studied production of BDO from whey by *K. pneumoniae* ATCC 13882 [23]. After 60 h of fermentation, 19.3 g/L BDO was produced from whey with a productivity of 0.32 g/L/h. Ramachandran et al. obtained a concentration of 32.49 g/L BDO from lactose by using *K. oxytoca* ATCC 8724; however, the yield (0.207 g/g lactose) and productivity (0.861 g/L/h) of BDO were still unsatisfactory [31]. In a previous work, *Lactococcus lactis* MG1363 was metabolically engineered to produce BDO from residual whey permeate, and a final titer of 51 g/L BDO was acquired [32]. Exogenous antibiotics were needed for the maintenance of two plasmids, pJM001 and pLP712, which carry the genes needed for BDO production and metabolism of lactose, respectively. To make bio-based BDO production from whey more economically efficient and environment-friendly, BDO production without antibiotic addition to the fermentation system for the maintenance of plasmids should be initiated. In this work, *K. oxytoca* PDL-0 was metabolically engineered to efficiently produce BDO from lactose in whey powder through deleting *pox*, *pta*, *frdA*, *ldhD*, and *pflB*. Using whey powder as the carbon source, the recombinant strain *K. oxytoca* PDL-K5 can produce 65.5 g/L BDO (Table 1). Compared with other strains used for BDO production from whey, the engineered strain has significant production advantages, such as high product concentration (65.5 g/L), high productivity (2.73 g/L/h), and lack of a need for unnecessary exogenous antibiotics.

**Table 1:** Comparison of BDO production using whey/lactose as substrate by different microorganisms

Strain	Substrate	Method	Concentration (g/L)	Yield (g/g)	Productivity (g/L/h)	References
*Bacillus polymyxa* ATCC 1232	Cheese whey	Wild-type	5.5	0.25	0.03	[22]
*K. pneumoniae* NCIB 8017	Rennet whey permeate	Wild-type	7.5	0.46	0.08	[42]
*K. oxytoca* NRRL-13-199	Whey	Wild-type, adding 50 mM acetate	8.4	0.365	–	[30]
*Enterobacter aerogenes* 3889	Whey	Wild-type, using neutralized acid whey with 50 mM acetate	15.1	–	0.24	[23]
*K. pneumoniae* ATCC 13882	Whey	Wild-type, using unsterilized acid whey and adjusting pH to 6.5	19.3	–	0.32	[23]
*Lactococcus lactis* mL001	Residual whey permeate (lactose)	Deletion of *ldh*, *ldhB*, *ldhX*, *pta*, *adhE*, *butBA*, overexpression of *bdh* and lactose utilizing pathway in *L. lactis* MG1363	51	0.47	1.46	[32]
*K. oxytoca* PDL-K5	Whey powder	Deletion of *pox*, *pta*, *frdA*, *ldhD, pflB* in *K. oxytoca* PDL-0	65.5	0.44	2.73	This study
*K. pneumoniae* KG1	Lactose	Wild-type	4.38	0.33	0.365	[21]
*K. oxytoca* NRRL-B199 with nonviable cells of *Kluyveromyces lactis* CBS 683	Lactose	Wild-type, co-immobilization by adhesion of β-galactosidase in nonviable cells of *K. lactis* with *K. oxytoca*	14.3	0.29	0.80	[25]
*K. oxytoca* ATCC 8724	Lactose	Wild-type	32.49	0.207	0.861	[31]
*K. oxytoca* PDL-K5	Lactose	Deletion of *pox*, *pta*, *frdA*, *ldhD, pflB* in *K. oxytoca* PDL-0	74.9	0.43	2.27	This study

**Fig. 6:**
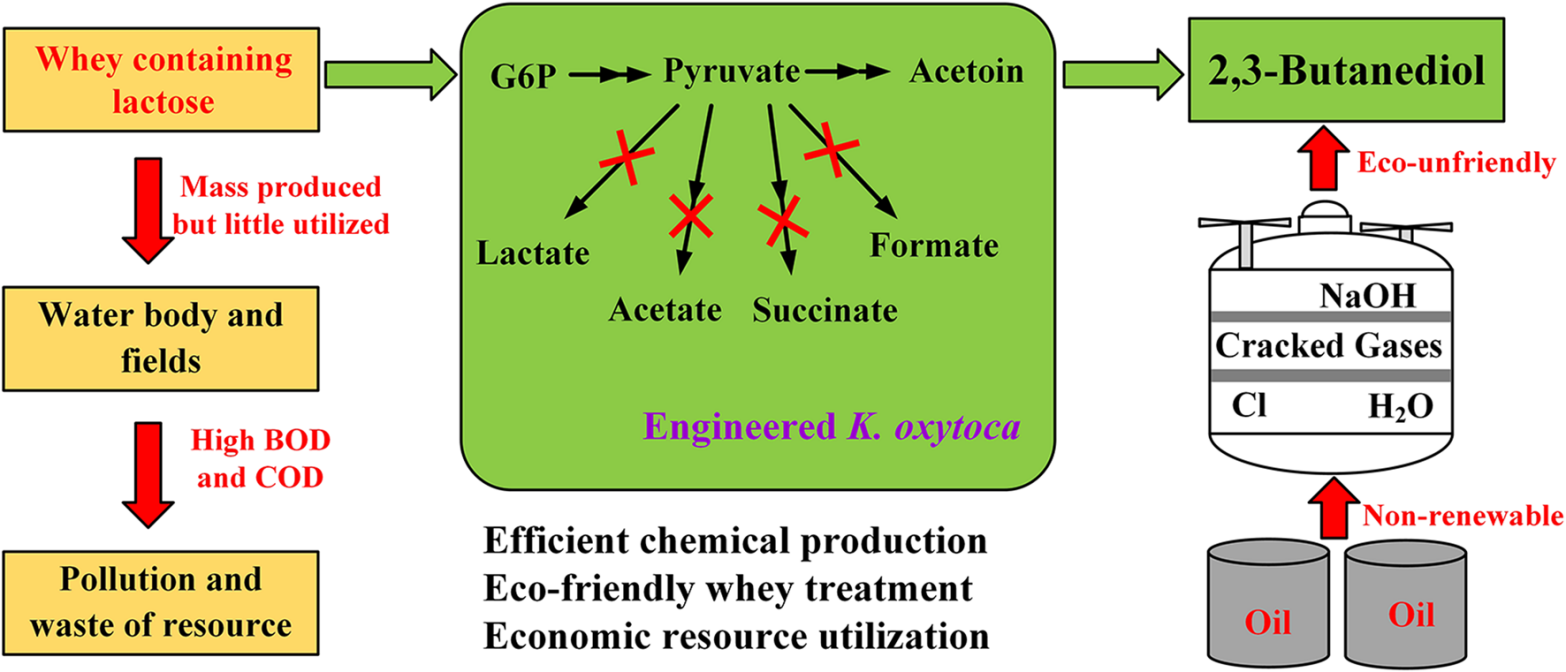
Scheme of BDO production from whey using metabolic engineered *K. oxytoca*. *G6P* glucose-6-phosphate

Recently, lactose or whey have been used to produce various biochemicals, e.g., ethanol [33], butanol [34], lactic acid [35], citric acid [36], poly(3-hydroxybutyrate) (PHB) [37], and gluconic acid [38], through endogenous or exogenous biosynthetic pathways. However, because of the low utilization efficiency of lactose in these chassis cells, it is difficult to produce the target chemicals with high productivity and high yield [34, 36]. Ahn et al. constructed a fermentation strategy with a cell-recycle membrane system for the production of PHB from whey [37]. A high consumption rate of lactose (7.67 g/L/h) was acquired using this complicated fermentation strategy. The engineered strain *K. oxytoca* PDL-K5 in this study had the ability to efficiently transform lactose in whey powder into BDO with relatively high yield (0.44 g/g) and high consumption rate of lactose (6.18 g/L/h). This work provides a suitable method for BDO production as well as whey utilization (Fig. 6). Considering its excellent characteristics of non-pathogenicity (Risk Group 1) and efficient lactose utilization, *K. oxytoca* PDL-0 may be a promising chassis for production of various chemicals from whey through metabolic engineering. For example, acetoin, the oxidized precursor of BDO, might be produced through increasing dissolved oxygen levels and deleting 2,3-butanediol dehydrogenases responsible for BDO production from acetoin [39].

## Conclusions

In this study, the ability of *K. oxytoca* PDL-0 to metabolize lactose and produce BDO was identified. Then, by-product pathways encoding genes in *K. oxytoca* PDL-0 were knocked out to improve the yield of BDO. The engineered strain *K. oxytoca* PDL-K5 was able to utilize whey powder as the substrate for high production of BDO. The fermentative process developed here is a promising alternative method for both biotechnological production of BDO and whey utilization. In addition, other important chemicals may also be produced from whey using metabolically engineered *K. oxytoca* PDL-0, which has the characteristics of efficient lactose utilization.

## Methods

### Enzymes and chemicals

FastPfu DNA polymerase was purchased from TransGen Biotech (Beijing, China) and T4 DNA ligase from Thermo Scientific (Lithuania). Restriction enzymes were purchased from TaKaRa Bio Inc. (Dalian, China). Polymerase chain reaction (PCR) primers were provided by Tsingke Biology Co., Ltd (QingDao, China). Racemic acetoin and BDO was purchased from Apple Flavor & Fragrance Group (Shanghai, China) and ACROS (The Kingdom of Belgium), respectively. Whey powder with a lactose content of 77% was purchased from KuoQuan Biotech (Shandong, China). All other chemicals were of analytical grade and commercially available.

### Bacterial strains, plasmids and culture medium

The strains and plasmids used in this study are listed in Table 2. All engineered strains used in this work are based on *K. oxytoca* PDL-0 and its derivatives. *E. coli* S17-1 was used to hold and amplify plasmids as well as for conjugation with *K. oxytoca*. The plasmid pKR6K_Cm_ was used for gene knockout in *K. oxytoca* [27].

**Table 2:** Strains and plasmids used in this study

Strain or plasmid	Characteristic(s)	References or sources
Strain		
*Escherichia coli* S17-1	*recA*, *pro*, *thi*, conjugative strain able to host λ-pir-dependent plasmids	[43]
*Enterobacter cloacae* SDM	Wild-type	[12]
*E. coli* BL21-pETRABC	*E. coli* BL21 (DE3) harboring pET-RABC	[26]
*Klebsiella pneumonia* ATCC 15380	Wild-type	ATCC
*Bacillus licheniformis* DSM13	Wild-type	DSMZ
*Klebsiella oxytoca* PDL-0	Wild-type	CCTCC M 2016184
*K. oxytoca* PDL-K1	*K. oxytoca* PDL-0 with deletion of *pox*	This study
*K. oxytoca* PDL-K2	*K. oxytoca* PDL-0 with deletion of *pox* and *pta*	This study
*K. oxytoca* PDL-K3	*K. oxytoca* PDL-0 with deletion of *pox, pta*, and *frdA*	This study
*K. oxytoca* PDL-K4	*K. oxytoca* PDL-0 with deletion of *pox, pta*, *frdA*, and *ldhD*	This study
*K. oxytoca* PDL-K5	*K. oxytoca* PDL-0 with deletion of *pox, pta*, *frdA*, *ldhD*, and *pflB*	This study
Plasmid		
pKR6K_Cm_	Cm^r^, gene replacement vector derived from plasmid pK18*mobsacB*, R6K origin, Mob^+^ *sac*B, and the Km^r^ resistance was replaced by Cm^r^	[27]
pKDΔ*pox*	pKR6K_Cm_ derivative, carries a 580 bp deletion of *pox*	This study
pKDΔ*pta*	pKR6K_Cm_ derivative, carries a 1152 bp deletion of *pta*	This study
pKDΔ*frdA*	pKR6K_Cm_ derivative, carries a 720 bp deletion of *frdA*	This study
pKDΔ*ldhD*	pKR6K_Cm_ derivative, carries a 386 bp deletion of *ldhD*	This study
pKDΔ*pflB*	pKR6K_Cm_ derivative, carries a 1150 bp deletion of *pflB*	This study

Luria–Bertani (LB) medium was used for the cultivation of all the strains used. The M9 minimal medium [40] supplemented with 5 g/L yeast extract and 40 g/L lactose was used in shake flasks experiments for selection of the efficient BDO producing strain. The selection medium for single exchange strains of *K. oxytoca* was M9 minimal medium supplemented with 20 g/L sodium citrate and 40 µg/mL chloramphenicol. The selection medium for double exchange strains of *K. oxytoca* was solid LB medium supplemented with 15% sucrose.

### Knockout the genes of *K. oxytoca* PDL-0

The primers used for knockout of byproduct-producing genes in *K. oxytoca* PDL-0 are listed in Additional file 1: Table S1. Vector isolation, restriction enzyme digestion, agarose gel electrophoresis, and other DNA manipulations were carried out using standard protocols [41]. Knockout mutants of *K. oxytoca* PDL-0 were generated via allele exchange using the suicide plasmid pKR6K_Cm_ [27]. The left and right flanking sequences were amplified from *K. oxytoca* PDL-0 and then ligated through PCR to get Δ*pox* fragment using primer pairs PΔ*pox*.f (EcoRI)/PΔ*pox*.r (overlap) and PΔ*pox*.f (overlap)/PΔ*pox*.r (BamHI), respectively. The gel-purified Δ*pox* fragments were ligated to the pKR6K_Cm_ digested with EcoRI and BamHI. The resulting plasmid was designated pKDΔ*pox* and introduced into *E. coli* S17-1. Then, a three-step deletion procedure was applied to select the Δ*pox* mutant after conjugating the pKDΔ*pox* in *K. oxytoca* PDL-0 as described previously [27]. The *pta*, *frdA*, *ldhD*, and *pflB* mutants of strain *K. oxytoca* PDL-0 were generated by using the same procedure and primers listed in Additional file 1: Table S1.

### Batch and fed-batch fermentations

Batch fermentations were conducted in a 1-L bioreactor (Multifors 2, Infors AG, Switzerland) with 0.8 L of medium. The seed culture was inoculated (10%, v/v) into the fermentation medium containing 8.27 g/L corn steep liquor powder (CSLP); 4.91 g/L (NH_4_)_2_HPO_4_; 3 g/L sodium acetate; 0.4 g/L KCl; 0.1 g/L MgSO_4_; 0.02 g/L FeSO_4_·7H_2_O; 0.01 g/L MnSO_4_·7H_2_O and 40 g/L lactose. The cultivation was carried out at 37 °C, stirring at 400 rpm, airflow at 1.0 vvm and initial pH of 7.0. When pH dropped to 6.0, it was maintained at this level by automatic addition of 4 M H_3_PO_4_ or 5 M NaOH. Fed-batch fermentation was carried out in a 7.5-L fermenter (BioFlo 310, NBS, USA) containing 5 L of medium and the cultivation condition was the same as 1-L fermenter except that the initial concentration of lactose was about 100 g/L. Alternatively, 130 g/L whey powder was fed into the fermentation broth to make the initial concentration of lactose at about 100 g/L. Solid lactose or whey powder was fed in the fermenter when residual lactose concentration was reduced to about 20 g/L.

### Analytical methods

The optical density (OD) was measured at 600 nm using a spectrophotometer (V5100H, Shanghai Metash Instruments Co., Ltd, China) after an appropriate dilution. The concentrations of lactose and other by-products were detected by high performance liquid chromatography (HPLC) in an Agilent 1100 series, equipped with a Aminex HPX-87H column (300 × 7.8 mm; Bio-Rad, USA) and a refractive index detector [40]. The mobile phase was 10 mM H_2_SO_4_ at a flow rate of 0.4 mL/min at 55 °C. The concentrations of acetoin and BDO were analyzed by gas chromatography (GC) (Shimadzu, GC2014c) using a capillary GC column (AT. SE-54, inside diameter, 0.32 mm; length, 30 m, Chromatographic Technology Center, Lanzhou Institute of Chemical Physics, China). Prior to GC analysis, the sample was extracted by ethyl acetate with isoamyl alcohol as the internal standard. Nitrogen was used as the carrier gas for GC analysis. The temperature of both the injector and the detector was 280 °C, the column oven was maintained at 80 °C for 3 min. Statistical analysis of the results was conducted using Origin 9.0 (OriginLab, USA). Unless otherwise specified, data are shown as the mean ± S.D. (standard deviations) from three independent experiments.

## Supplementary information

**Additional file 1.** Experimental detail (Table S1, Figure S1) and Additioanl data (Figure S2).

## Data Availability

All data generated or analyzed during this study are included in this published article and its additional file.
