# Supplementary material for: Efficient 2,3-butanediol production from whey powder using metabolically engineered Klebsiella oxytoca
Source: Microb Cell Fact. 2020 Aug 10;19:162. doi: 10.1186/s12934-020-01420-2 (PMC7419187; doi:10.1186/s12934-020-01420-2)
Supplement: Supplementary file 1 — Additional file 1. Experimental detail (Table S1, Figure S1) and Additioanl data (Figure S2). [file 12934_2020_1420_MOESM1_ESM.docx]

***Supporting Information***

**Efficient 2,3-butanediol production from whey powder using metabolically engineered *Klebsiella oxytoca***

Wensi Meng^1^, Yongjia Zhang^1^, Menghao Cao^1^, Wen Zhang^2^, Chuanjuan Lü^1*^, Chunyu Yang^1^, Chao Gao^1^, Ping Xu^3^, Cuiqing Ma^1*^

^1^*State Key Laboratory of Microbial Technology, Shandong University, Qingdao 266237, People’s Republic of China*

^2^*Center for Gene and Immunotherapy, The Second Hospital of Shandong University, Jinan 250033, People’s Republic of China*

^3^*State Key Laboratory of Microbial Metabolism, Joint International Research Laboratory of Metabolic & Developmental Sciences, and School of Life Sciences & Biotechnology, Shanghai Jiao Tong University, Shanghai 200240, People’s Republic of China*

*Corresponding authors:

Mailing address for C. Lü: State Key Laboratory of Microbial Technology, Shandong University, Qingdao 266237, People’s Republic of China, Tel.: 86-532-58631561, Fax: +86-532-58631561, E-mail: chuanjuanlv@mail.sdu.edu.cn.

Mailing address for C. Ma: State Key Laboratory of Microbial Technology, Shandong University, Qingdao 266237, People’s Republic of China, Tel.: 86-532-58631561, Fax: +86-532-58631561, E-mail: [macq@sdu.edu.cn](mailto:macq@sdu.edu.cn).

**Table S1** Sequences of primers used in this study.

| Primer^a^ | Sequence (5′-3′)^b^ |
| --- | --- |
| PΔ*pox*.f (EcoRI) | CCGGAATTCACAGACCGTGGCGGCATACA |
| PΔ*pox*.r (overlap) | CGCTTACCGTTCATCTGCAAAGCTGGGCCAGCTTTTTCAG |
| PΔ*pox*.f (overlap) | CTGAAAAAGCTGGCCCAGCTTTGCAGATGAACGGTAAGCG |
| PΔ*pox*.r (BamHI) | CGCGGATCCTTACCTTAGCCAGTTAGTT |
| PΔ*pta*.f (EcoRI) | CCGGAATTCACTGGCGGTAACGAAAGAGGATA |
| PΔ*pta*.r (overlap) | TAAACCTGTTCCGGCAGCACGAAGCTGCTGCGAGTCAG |
| PΔ*pta*.f (overlap) | CTGACTCGCAGCAGCTTCGTGCTGCCGGAACAGGTTTA |
| PΔ*pta*.r (XbaI) | TGCTCTAGATTATGCTTGCTGCTGGGACGAC |
| PΔ*frdA*.f (EcoRI) | CCGGAATTCATACCGTTGCTGCTGAAGGG |
| PΔ*frdA*.r (overlap) | CTTCGCCCAGTTCTCGTTACTGGTATTGTAGCGATACACG |
| PΔ*frdA*.f (overlap) | CGTGTATCGCTACAATACCAGTAACGAGAACTGGGCGAAG |
| PΔ*frdA*.r (BamHI) | CGCGGATCCTCAGCCATTCGTCGTCTC |
| PΔ*ldhD*.f (EcoRI) | CCGGAATTCTACGAAACAGTACGACAAG |
| PΔ*ldhD*.r (overlap) | GAATCGATGAGCGCGCCGCACCGCTTCCGGCGAGTAGGC |
| PΔ*ldhD*.f (overlap) | GCCTACTCGCCGGAAGCGGTGCGGCGCGCTCATCGATTC |
| PΔ*ldhD*.r (BamHI) | CGCGGATCCTGACGCAGGTTGTCGAGGGT |
| PΔ*pflB*.f (EcoRI) | CCGGAATTCTTAATGAAAAGTTAGCCACA |
| PΔ*pflB*.r (overlap) | CGCGAGAGTCGTTGTTACCAGACGGTAGTCACCGATGATA |
| PΔ*pflB*.f (overlap) | TATCATCGGTGACTACCGTCTGGTAACAACGACTCTCGCG |
| PΔ*pflB*.r (XbaI) | TGCTCTAGATTACATGGTCTGAGTGAAGG |

^a^f indicates that this is a sense primer; r indicates that this is an antisense primer.

^b^Restriction sites are underlined.

**
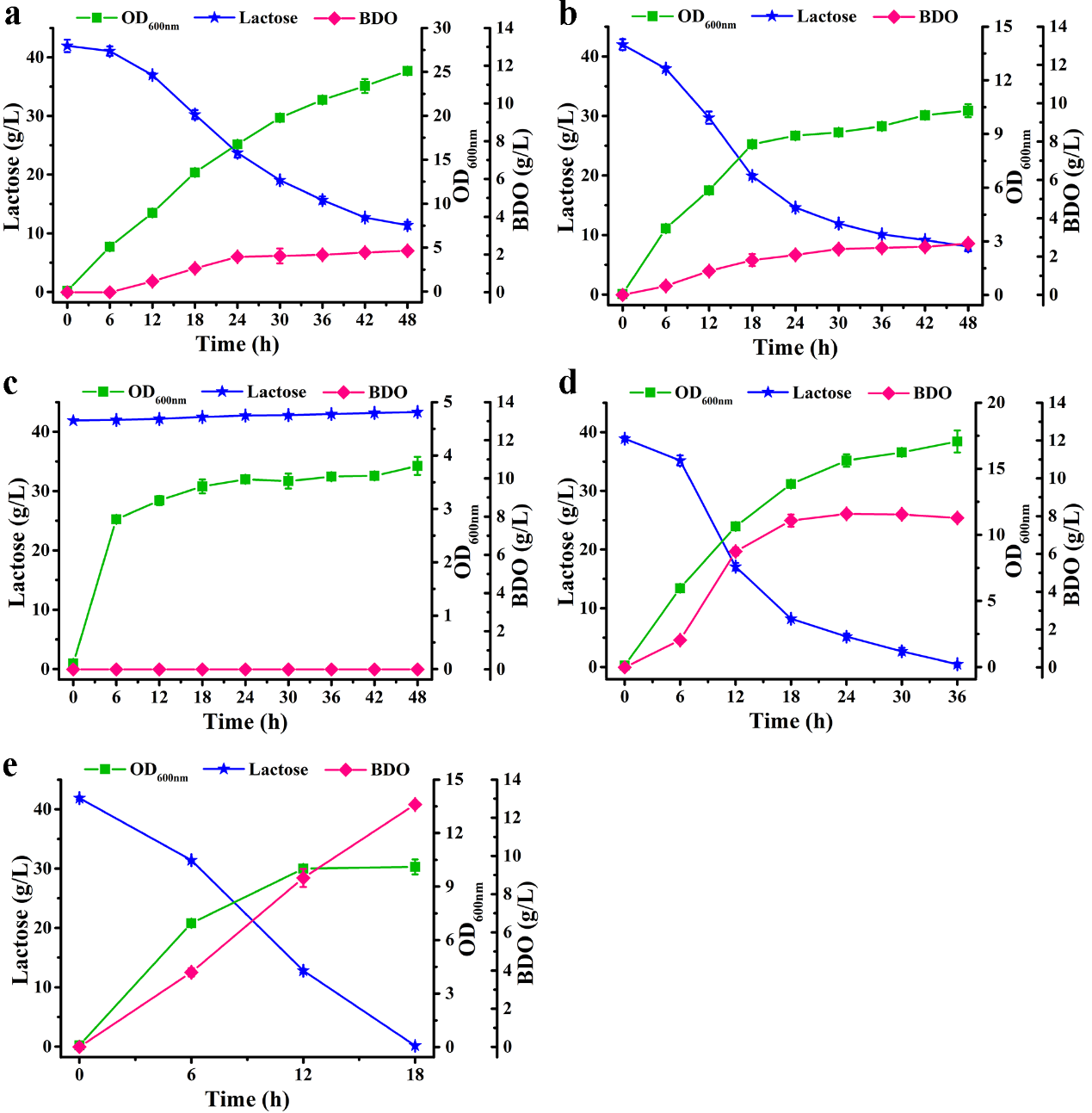
**

**Fig. S1** Time profiles of growth, lactose consumption and BDO production of *E. cloacae* SDM (a), *E. coli* BL21-pETRABC (b), *B. licheniformis* DSM13 (c), *K. pneumonia* ATCC 15380 (d), *K. oxytoca* PDL-0 (e). Data shown are mean ± s.d. (n = 3 independent experiments).

**
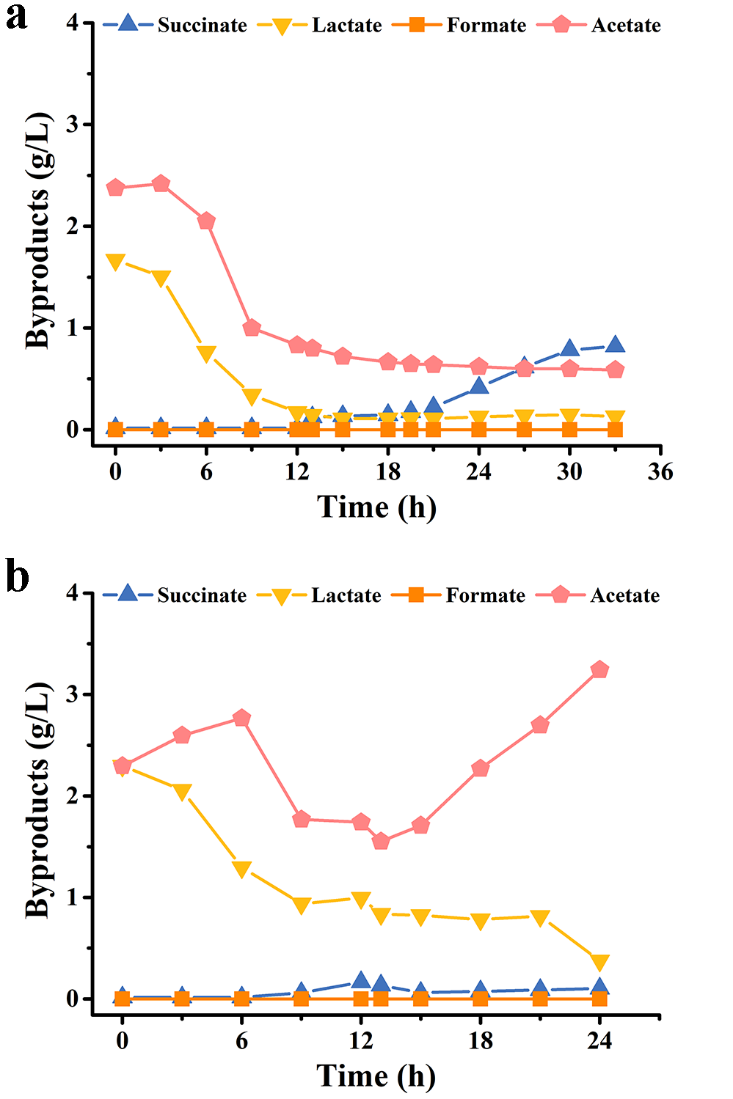
**

**Fig. S2** By-products of fed-batch fermentation using lactose (a) and whey powder (b) as the carbon source. Concentration of succinate, lactate, formate and acetate by *K. oxytoca* PDL-K5 were assayed. The experiments were conducted in a 7.5-L fermenter containing 5 L of medium with an initial lactose concentration of 100 g/L approximately. Cultivation was carried out at an initial pH of 7.0 and maintained at 6.0 when it dropped to 6.0 by automatic addition of 4 M H_3_PO_4_ or 5 M NaOH. The agitation speed was 400 rpm and the aeration rate was 1 vvm.
